# Supplementary material for: Protocol: Effects of midazolam on postoperative delirium in elderly patients undergoing spinal surgery: A randomized, double-blind, placebo-controlled non-inferiority trial
Source: PLoS One. 2026 Feb 6;21(2):e0339537. doi: 10.1371/journal.pone.0339537 (PMC12880649; doi:10.1371/journal.pone.0339537)
Supplement: S2 File — (DOC) [file pone.0339537.s002.doc]

咪达唑仑对老年脊柱手术患者术后谵妄的影响：

前瞻、双盲、随机对照研究

1. **研究背景**

术后谵妄（ POD） 是术后急性脑功能障碍，发生率 1.4% ~60%，其中老年患者发生率为15~53%，是老年手术患者的常见并发症；POD延长住院时间、增加其他术后并发症、增加认知能力障碍、痴呆甚至死亡的发生率；增加医疗费用、家庭及社会负担[1]。随着全球进入老龄化社会，有关老年手术患者POD的研究关注度日益增高[2]。咪达唑仑是最常用的静脉全麻诱导药物，具有镇静、催眠、抗焦虑、顺行性遗忘等作用，在临床上的应用极为广泛。在全身麻醉前，咪达唑仑常用于诱导镇静状态，有效缓解患者的术前焦虑，为手术提供一个平稳的起始状态，并且对血液动力学的影响小，有助于降低术后恶心呕吐（PONV）及术中知晓的发生率[3]。此外，它在急诊科、重症监护室以及内镜检查等多种医疗场合中也扮演着重要角色，帮助患者快速进入适当的镇静状态，以便于进行各种诊疗操作。因此围术期咪达唑仑扔被常规使用。以往研究发现，术后谵妄与咪达唑仑的应用有关[4]，咪达唑仑是导致住院患者，特别是ICU患者谵妄的药物之一，咪达唑仑用于ICU患者的深度镇静时,7d内谵妄的发生率高达152/207(73%) [5,6]，美国老年医学学会和其他指南都曾建议老年患者手术中尽量减少咪达唑仑的使用[7]。但新进研究发现围术期应用咪达唑仑并不导致POD[8]。该研究中发现咪达唑仑在围手术期的使用与年轻的成人患者发生术后谵妄风险的增加无关，但针对老年患者的随机研究数量有限，因此需要更多针对老年患者的研究来确认这一发现。一项大型多中心随机对照试验评估咪达唑仑术前给药对老年外科手术患者预后的影响以及术中使用苯二氮卓类药物对心脏麻醉患者谵妄的影响的结果目前尚未确定[9]。综上所述，鉴于咪达唑仑在全麻诱导中的重要作用，明确咪达唑仑对老年患者POD的影响具有重要临床意义。

1. **研究目的**

本研究通过前瞻、双盲、随机对照研究，明确2mg咪达唑仑静脉全麻诱导对老年脊柱手术患者POD的影响

1. **研究设计**

**3.1整体的研究设计和计划**

预期目标：通过随机对照研究方法，比较静脉全麻诱导时咪达唑仑使用与否对择期老年脊柱手术患者POD发生率的影响，为咪达唑仑的临床合理应用提供依据。

研究方案：研究方案报医院伦理委员会同意，并在“中国临床试验注册中心（http://www.chictr.org/Default.aspx）”注册登记。研究属于前瞻、双盲、随机对照研究。

**3.2研究人群**

2025年10月1日~2026年4月1日期间宁波市第六医院择期老年（年龄≥65岁）脊椎手术患者，男女不限

**3.2.1入选标准**

1)年龄65-90岁；

2)计划实行全身麻醉的脊柱手术；

3)美国麻醉医师协会的身体状况（ASA）I-III类；

4)同意参加研究并已签署知情同意书。

如果患者符合所有的纳入标准，则被纳入研究

**3.2.2排除标准**

1）美国麻醉医师协会（ASA）Ⅴ级及以上；

2）术前入住ICU ，和/或术后因病情因素转入ICU者；

3）多部位骨折手术

4）未经治疗或治疗不足的甲亢患者

5）有严重心血管疾病患者（如六个月内发生心梗，不稳定性心绞痛和充血性心力衰竭）

6）眼压增高患者（如青光眼）

7）无法正常交流或存有严重的精神类疾病患者

8）颅内压增高患者

9）术前经MMSE评估，属于中重度痴呆患者

10）拒绝签署研究知情同意书

**3.3.3：病例数及分组方法**

根据已报道的数据，接受脊椎手术的老年人中POD发生率在0.84%至24.6%之间[17-19]。结合类似的研究设计[20，21]，我们将非劣效性界限设定为9%。我们的预实验数据显示，咪达唑仑组的POD发生率约为20%，与安慰剂组相当。利用这些数据，我们使用PASS 15.0.5版软件计算了样本量。采用Z检验（合并方差），单侧α值为0.025，功效为0.8，计算得出每组所需的样本量为311例，共622例受试者。

1. **研究步骤**

**4.1 研究地点和类型：**本研究是随机对照实验，在宁波市第六医院麻醉科和骨科进行。所有符合条件且知情的患者在随机分组前均提供了签署的书面知情同意书。

**4.2 干预措施：**符合条件的参与者622名患者按1∶1的比例采用计算机随机分组，每组311人，试验组接受2mg咪达唑仑静脉诱导，对照组接受同等剂量的生理盐水静脉注射诱导，为了隐藏分配任务，药物被放置在密封的不透明信封中，随机分组后，在进入手术室前依次交给麻醉医生。参与研究的麻醉医生知道患者的分组情况，但他们没有参与随访评估。随访调查人员对干预措施并不知情。受试者入手术室后开通静脉通路，常规监测心电图、无创血压和连续氧饱和度，在手术过程中，用双谱指数（BIS）监测麻醉深度。两组患者给予咪达唑仑（2mg）或同等剂量生理盐水，丙泊酚（2-3mg kg−1），舒芬太尼（0.2ug kg−1），罗库溴铵（0.1 mg kg−1）进行麻醉诱导，通过调整丙泊酚剂量，使BIS值达到在40-60之间，然后进行气管插管。术中维持药物丙泊酚和瑞芬太尼。所有药物均在手术结束时停止输注，受试者手术结束后转入PACU进行复苏。所有参与者在手术结束前接受32mg昂丹司琼和布托啡诺12mg止痛泵减轻重度术后疼痛。麻醉医生在全身麻醉期间避免使用氯胺酮、右美托咪定和长托宁。术后进行随访评估。

**4.3：主要结局：**PACU术后躁动的发生率及术后谵妄评估，以RASS（Richmond躁动-镇静评分）评估患者拔管后躁动情况。以意识模糊评估法（Confusion Assessment Method，CAM）评估患者术后3天内谵妄发生情况。次要结局：患者的疼痛评分及不良事件的发生率，以视觉模拟评分法（Visual Analogue Scale，VAS）评估患者的疼痛程度。

**4.4：技术路线图**

**
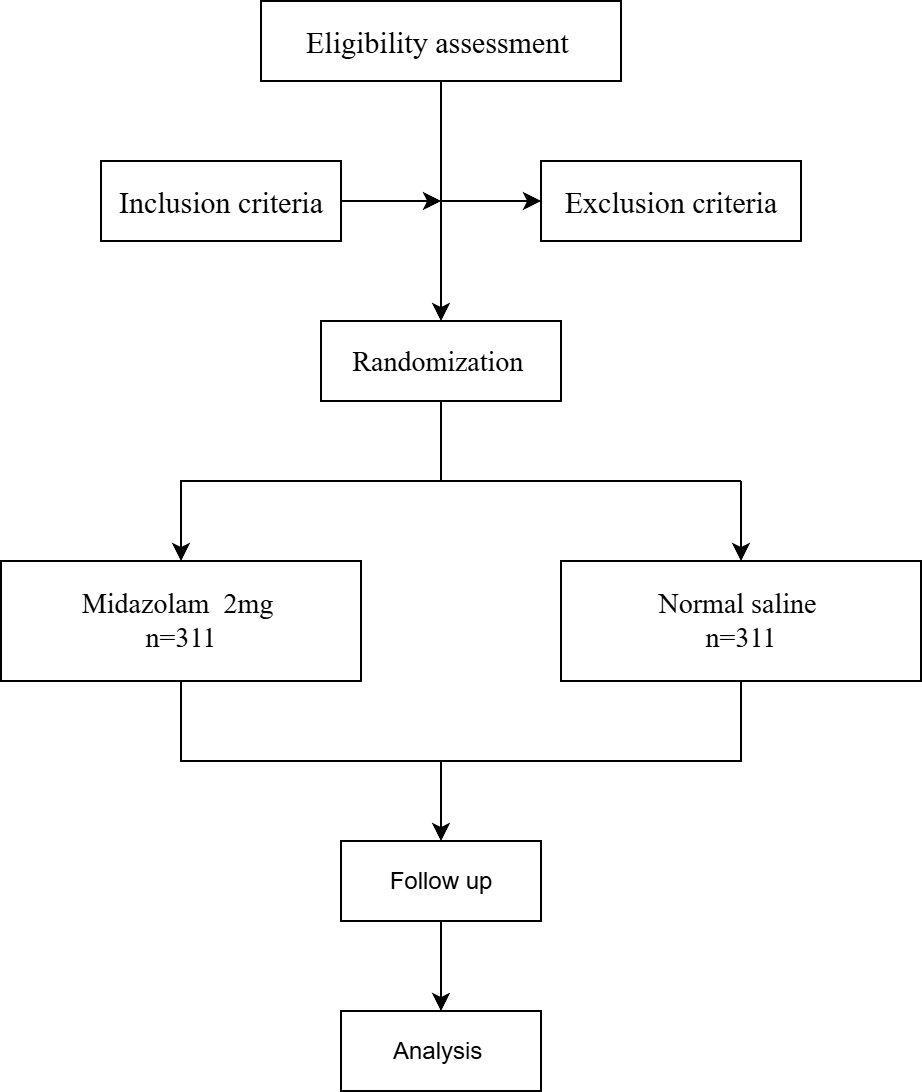
**

1. **统计分析**

数据使用 SPSS 19.0 版软件进行处理的。对于连续就，使用Shapiro–Wilk检验来检验其正态性。正态分布的数据以均值±标准差（SD）的形式报告，并使用 t 检验来分析差异。非正态分布的数据则使用Mann–Whitney U检验进行分析。对于分类数据，使用Fisher's精确检验或卡方（χ²）检验进行分析。通过逻辑回归对 POD 发生的潜在影响因素进行了多变量分析。当 P < 0.05 时，认为存在统计学显著差异。

1. **研究相关伦理学**

**6.1伦理委员会的审核**

本研究方案及与患者相关的资料必须提交伦理委员会的审核，获得伦理委员会书面同意之后方可开展。

1. **保密措施** 本研究的结果可能会在医学杂志上发表，但是我们会按照法律的要求为患者的信息保密，患者的个人信息不会被泄露。必要时，政府管理部门和医院伦理委员会及其有关人员可以按规定查阅患者的资料。
2. **、研究的预期进度和完成日期**

研究时限为2025年10月1日至2026年5月1日

1. **参考文献**

1. O, d.l.V.-M., et al., *Development and validation of a delirium risk prediction preoperative model for cardiac surgery patients (DELIPRECAS): An observational multicentre study.* Journal of clinical anesthesia, 2021. **69**: p. 110158.

2. Huang, J.-X., et al., *The role of perioperative sedative anesthetics in preventing postoperative delirium: a systematic review and network-meta analysis including 6679 patients.* BMC Cardiovascular Disorders, 2024. **24**(1).

3. E, A., et al., *The effect of perioperative benzodiazepine administration on postoperative nausea and vomiting: a systematic review and meta-analysis of randomised controlled trials.* British journal of anaesthesia, 2024. **132**(3): p. 469-482.

4. MS, A., et al., *Pain, fentanyl consumption, and delirium in adolescents after scoliosis surgery: dexmedetomidine vs midazolam.* Paediatric anaesthesia, 2013. **23**(5): p. 446-52.

5. TG, v.G., et al., *The risk of delirium after sedation with propofol or midazolam in intensive care unit patients.* British journal of clinical pharmacology, 2024. **90**(6): p. 1471-1479.

6. Spence, J., et al., *Benzodiazepine-Free Cardiac Anesthesia for Reduction of Postoperative Delirium (B-Free): A Protocol for a Multi-centre Randomized Cluster Crossover Trial.* CJC Open, 2023. **5**(9): p. 691-699.

7. JW, D., et al., *Clinical Practice Guidelines for the Prevention and Management of Pain, Agitation/Sedation, Delirium, Immobility, and Sleep Disruption in Adult Patients in the ICU.* Critical care medicine, 2018. **46**(9): p. e825-e873.

8. E, W., et al., *Effect of perioperative benzodiazepine use on intraoperative awareness and postoperative delirium: a systematic review and meta-analysis of randomised controlled trials and observational studies.* British journal of anaesthesia, 2023. **131**(2): p. 302-313.

9. VJ, L., et al., *Perioperative benzodiazepine administration among older surgical patients.* British journal of anaesthesia, 2021. **127**(2): p. e69-e71.

10. E, Y., et al., *Association between postoperative delirium and adverse outcomes in older surgical patients: A systematic review and meta-analysis.* Journal of clinical anesthesia, 2023. **90**: p. 111221.
