# Supplementary material for: Protocol: Effects of midazolam on postoperative delirium in elderly patients undergoing spinal surgery: A randomized, double-blind, placebo-controlled non-inferiority trial
Source: PLoS One. 2026 Feb 6;21(2):e0339537. doi: 10.1371/journal.pone.0339537 (PMC12880649; doi:10.1371/journal.pone.0339537)
Supplement: S3 File — (PDF) [file pone.0339537.s003.pdf]

The Impact of Midazolam on Postoperative Delirium in Elderly Patients Undergoing Spinal Surgery:

Prospective, Double-Blind, Randomized Controlled Study

Research Background

Postoperative delirium (POD) is an acute brain dysfunction occurring after surgery, with an incidence rate ranging from 1.4% to 60%. Among elderly patients, the incidence rate is 15% to 53%, making it a common complication in elderly surgical patients. POD prolongs hospital stay, increases the risk of other postoperative complications, cognitive impairment, dementia, and even mortality; it also increases medical expenses and the burden on families and society [1]. With the global population aging, research on POD in elderly surgical patients has garnered increasing attention [2]. Midazolam is the most commonly used intravenous anesthetic induction drug, with effects including sedation, hypnosis, anti-anxiety, and anterograde amnesia, making it widely used in clinical practice. Before general anesthesia, midazolam is often used to induce a sedative state, effectively alleviating preoperative anxiety in patients and providing a stable starting point for surgery, while having a minimal impact on hemodynamics, which helps reduce the incidence of postoperative nausea and vomiting (PONV) and intraoperative awareness [3]. Additionally, it plays a significant role in various medical settings such as emergency departments, intensive care units (ICUs), and endoscopic examinations, helping patients quickly enter an appropriate sedative state for various diagnostic and therapeutic procedures. Therefore, midazolam remains routinely used in the perioperative period. Previous studies have found that POD is associated with the use of midazolam [4], and midazolam is one of the drugs associated with delirium in hospitalized patients, particularly ICU patients. When used for deep sedation in ICU patients, the incidence of delirium within 7 days is as high as 152/207 (73%) [5,6]. The American Geriatric Society and other guidelines have previously recommended minimizing the use of midazolam in elderly patients during surgery [7]. However, recent studies have found that the use of midazolam during the perioperative period does not lead to POD [8]. This study found that the use of midazolam during the perioperative period is not associated with an increased risk of POD in young adult patients, but the number of randomized studies in elderly patients is limited. Therefore, more research targeting elderly patients is needed to confirm this finding. A large multicenter randomized controlled trial is currently underway to evaluate the impact of preoperative midazolam administration on the prognosis of elderly

surgical patients and the effect of benzodiazepine use during surgery on delirium in cardiac anesthesia patients [9]. In summary, given the important role of midazolam in anesthetic induction, it is clinically significant to clarify the impact of midazolam on POD in elderly patients.

Research Objective

This study aims to clarify the impact of 2mg midazolam intravenous general anesthesia induction on postoperative delirium (POD) in elderly patients undergoing spinal surgery through a prospective, double-blind, randomized controlled trial.

Research Design

3.1 Overall research design and plan

Expected Objective: To compare the effect of midazolam use versus non-use during intravenous general anesthesia induction on the incidence of postoperative delirium (POD) in elderly patients undergoing elective spinal surgery using a randomized controlled trial method, and to provide a basis for the clinical rational application of midazolam.

Research Protocol: The research protocol was approved by the hospital ethics committee and registered at the "China Clinical Trial Registry (http://www.chictr.org/Default.aspx)". The study is a prospective, double-blind, randomized controlled trial.

3.2 Research Population

Elderly patients (age ≥ 65 years) undergoing elective spinal surgery at Ningbo No. 6 Hospital from October 1, 2025, to April 1, 2026, regardless of gender.

3.2.1 Inclusion Criteria

1) Age 65-90 years;

2) Scheduled for spinal surgery under general anesthesia;

3) American Society of Anesthesiologists' physical status (ASA) I-III class;

4) Agreed to participate in the research and has signed the informed consent form.

If the patient meets all inclusion criteria, they will be enrolled in the study.

3.2.2 Exclusion Criteria

1) American Society of Anesthesiologists (ASA) Class V or higher;

2) Admission to the ICU preoperatively, and/or transfer to the ICU postoperatively due to clinical factors

3) Multifragmentary fracture surgery

4) Untreated or undertreated hyperthyroid patients

5) Patients with severe cardiovascular diseases (such as myocardial infarction within the past six months, unstable angina, and congestive heart failure)

6) Patients with elevated intraocular pressure (e.g., glaucoma)

7) Patients who cannot communicate normally or have severe mental illnesses

8) Patients with elevated intracranial pressure

9) Patients assessed preoperatively with MMSE, classified as moderate to severe dementia

10) Refusal to sign the research informed consent form

3.3.3: Number of cases and grouping method

Based on previously reported data, the incidence of POD in elderly patients undergoing spinal surgery ranges from 0.84% to 24.6% [17-19]. Combining similar study designs [20, 21], we set the non-inferiority margin at 9%. Our preliminary experimental data show that the POD incidence in the midazolam group was approximately 20%, comparable to the placebo group. Using this data, we calculated the sample size using PASS 15.0.5 software. Employing Z-test (pooled variance), a one-sided α of 0.025, and power of 0.8, the required sample size for each group was calculated as 311, totaling 622 participants.

Research steps

4.1 Research Site and Type: This study is a randomized controlled trial conducted in the Department of Anesthesiology and Orthopedics of the Sixth People's Hospital of Ningbo. All eligible and informed patients provided signed written informed consent before randomization.

4.2 Interventions: Among the 622 eligible participants, patients were randomly assigned in a 1:1 ratio using computer-generated randomization, with 311 patients in each group. The experimental group received intravenous midazolam (2mg) for induction, while the control group received intravenous saline injection of the same dose for induction. To conceal allocation tasks, the drugs were placed in sealed opaque envelopes and sequentially handed to anesthesiologists before entering the operating room. The anesthesiologists involved in the study were aware of the patients' group assignments but did not participate in follow-up assessments. Follow-up investigators were blinded to the interventions. After entering the operating room, intravenous access was established, and routine monitoring of electrocardiogram, non-invasive blood pressure, and continuous oxygen saturation was performed. During the surgery, anesthesia depth was monitored using the bispectral index (BIS). Both groups of patients received midazolam (2mg), propofol (2-3mg kg-1), sufentanil (0.2ug kg-1), and rocuronium (0.1 mg kg-1) for anesthesia induction. Propofol dosage was adjusted to achieve a BIS value between 40-60, followed by tracheal intubation. Propofol and remifentanil were maintained during the surgery. All medications were discontinued at the end of the surgery, and the participants were transferred to the Post-Anesthesia Care Unit (PACU) for recovery. All participants received a 32mg ondansetron and 12mg butorphanol PCA pump to alleviate severe postoperative pain before the end of the surgery.

Anesthesiologists avoided the use of ketamine, dexmedetomidine, and atropine during general anesthesia. Follow-up assessments were conducted after surgery.

4.3 Primary Outcomes: The incidence of post-anaesthesia care unit (PACU) postoperative agitation and postoperative delirium assessment. Postoperative agitation after extubation was evaluated using the Richmond Agitation-Sedation Scale (RASS). Postoperative delirium within 3 days was assessed using the Confusion Assessment Method (CAM). Secondary Outcomes: Patient pain scores and the incidence of adverse events, with pain intensity assessed using the Visual Analogue Scale (VAS).

4.4 Technical Roadmap

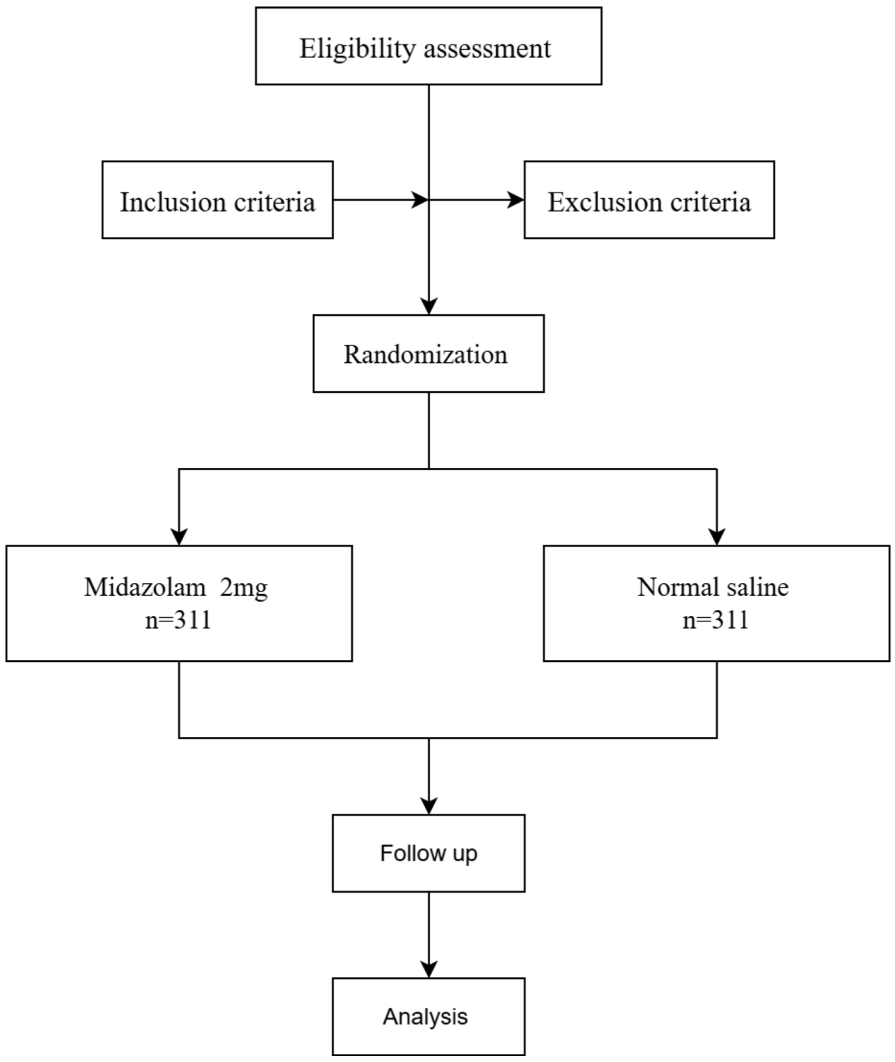

Statistical Analysis

Data were processed using SPSS 19.0 software. For continuous variables, the Shapiro‑Wilk test was used to test for normality. Normally distributed data were reported as mean ± standard deviation (SD) and analyzed using t-tests for differences. Non-normally distributed data were analyzed using the Mann‑Whitney U test. For categorical data, Fisher's exact test or chi-square (χ^2) test was used for analysis. Multivariate analysis was conducted using logistic regression to identify potential influencing factors of POD occurrence. A statistically significant difference was considered to exist when $P < 0.05$.

Ethical Considerations Related to the Study

6.1 Review by the Ethics Committee

This research proposal and patient-related materials must be submitted to the ethics committee for review, and written consent from the ethics committee must be obtained before the study can commence.

Confidentiality Measures The results of this study may be published in medical journals, but we will maintain patient confidentiality in accordance with legal requirements, and patient personal information will not be disclosed. When necessary, government regulatory authorities, hospital ethics committees, and their relevant personnel may access patient records in accordance with regulations.

Research Expected Timeline and Completion Date

The study period is from October 1, 2025, to May 1, 2026.

References

1. O, d.l.V.-M., et al., *Development and validation of a delirium risk prediction preoperative model for cardiac surgery patients (DELIPRECAS): An observational multicentre study.* Journal of clinical anesthesia, 2021. **69**: p. 110158.

2. Huang, J.-X., et al., *The role of perioperative sedative anesthetics in preventing postoperative delirium: a systematic review and network-meta analysis including 6679 patients.* BMC Cardiovascular Disorders, 2024. **24**(1).

3. E, A., et al., *The effect of perioperative benzodiazepine administration on postoperative nausea and vomiting: a systematic review and meta-analysis of randomised controlled trials.* British journal of anaesthesia, 2024. **132**(3): p. 469-482.

4. MS, A., et al., *Pain, fentanyl consumption, and delirium in adolescents after scoliosis surgery: dexmedetomidine vs midazolam.* Paediatric anaesthesia, 2013. **23**(5): p. 446-52.

5. TG, v.G., et al., *The risk of delirium after sedation with propofol or midazolam in intensive care unit patients.* British journal of clinical pharmacology, 2024. **90**(6): p. 1471-1479.

6. Spence, J., et al., *Benzodiazepine-Free Cardiac Anesthesia for Reduction of Postoperative Delirium (B-Free): A Protocol for a Multi-centre Randomized Cluster Crossover Trial.* CJC Open, 2023. **5**(9): p. 691-699.

7. JW, D., et al., *Clinical Practice Guidelines for the Prevention and Management of Pain, Agitation/Sedation, Delirium, Immobility, and Sleep Disruption in Adult Patients in the ICU.* Critical care medicine, 2018. **46**(9): p. e825-e873.

8. E, W., et al., *Effect of perioperative benzodiazepine use on intraoperative awareness and postoperative delirium: a systematic review and meta-analysis of randomised controlled trials and observational studies.* British journal of anaesthesia, 2023. **131**(2): p. 302-313.

9. VJ, L., et al., *Perioperative benzodiazepine administration among older surgical patients.* British journal of anaesthesia, 2021. **127**(2): p. e69-e71.

10. E, Y., et al., *Association between postoperative delirium and adverse outcomes in older surgical patients: A systematic review and meta-analysis.* Journal of clinical anesthesia, 2023. **90**: p. 111221.
