# Supplementary material for: Protocol: Effects of midazolam on postoperative delirium in elderly patients undergoing spinal surgery: A randomized, double-blind, placebo-controlled non-inferiority trial
Source: PLoS One. 2026 Feb 6;21(2):e0339537. doi: 10.1371/journal.pone.0339537 (PMC12880649; doi:10.1371/journal.pone.0339537)
Supplement: Attachment — Submitted filename: response 20251111.docx [file pone.0339537.s005.docx]

Dear Editors and Reviewers,

Thank you for giving me the opportunity to submit a revised draft of my manuscript titled *Protocol: Effects of midazolam on postoperative delirium in elderly patients undergoing spinal surgery: a randomized, double-blind, placebo-controlled non-inferiority trial* (PONE-D-25-37876) to PLOS ONE.

We appreciate the time and effort that you and the reviewers have dedicated to providing your valuable feedback on my manuscript. We are grateful to the reviewers for their insightful comments on our paper. We have incorporated changes to reflect most of the suggestions provided and have highlighted the revisions within the manuscript.

**Journal Requirements:**

When submitting your revision, we need you to address these additional requirements.

1. Please ensure that your manuscript meets PLOS ONE's style requirements, including those for file naming. The PLOS ONE style templates can be found at <https://journals.plos.org/plosone/s/file?id=wjVg/PLOSOne_formatting_sample_main_body.pdf> and <https://journals.plos.org/plosone/s/file?id=ba62/PLOSOne_formatting_sample_title_authors_affiliations.pdf.>

Response: Thank you for your guidance. We have revised the manuscript in strict accordance with the PLOS ONE style templates. Please feel free to point out any remaining inconsistencies or errors—we greatly appreciate your further feedback.

2. Thank you for stating in your Funding Statement: “This work was supported by Ningbo Clinical Research Center for Orthopedics, Sports Medicine & Rehabilitation (2024L004). The peer review comments of the funding body have not been made public, so we are unable to provide specific peer review opinions.” Please provide an amended statement that declares *all* the funding or sources of support (whether external or internal to your organization) received during this study, as detailed online in our guide for authors at <http://journals.plos.org/plosone/s/submit-now.> Please also include the statement “There was no additional external funding received for this study.” in your updated Funding Statement. Please include your amended Funding Statement within your cover letter. We will change the online submission form on your behalf.

Response: Thank you for the instruction. We have updated the Funding Statement as requested, including the required declaration about no additional external funding. The amended statement is included in the cover letter, and we kindly ask for your review.

3. PLOS requires an ORCID iD for the corresponding author in Editorial Manager on papers submitted after December 6th, 2016. Please ensure that you have an ORCID iD and that it is validated in Editorial Manager. To do this, go to ‘Update my Information’ (in the upper left-hand corner of the main menu), and click on the Fetch/Validate link next to the ORCID field. This will take you to the ORCID site and allow you to create a new iD or authenticate a pre-existing iD in Editorial Manager.

Response: We confirm that the ORCID iD has been successfully created, validated, and linked to the corresponding author’s profile in Editorial Manager.

4. Thank you for stating the following in the Acknowledgments Section of your manuscript: “This work was supported by Ningbo Clinical Research Center for Orthopedics, Sports Medicine & Rehabilitation (2024L004).” We note that you have provided additional information within the Acknowledgements Section that is not currently declared in your Funding Statement. Please note that funding information should not appear in the Acknowledgments section or other areas of your manuscript. We will only publish funding information present in the Funding Statement section of the online submission form. Please remove any funding-related text from the manuscript and let us know how you would like to update your Funding Statement. Currently, your Funding Statement reads as follows: “This work was supported by Ningbo Clinical Research Center for Orthopedics, Sports Medicine & Rehabilitation (2024L004).” Please include your amended statements within your cover letter; we will change the online submission form on your behalf.

Response: We sincerely appreciate your careful review and valuable guidance on our manuscript. In response to your comment regarding the funding information disclosure, we have completed the required revisions as follows:

1. All funding-related content has been thoroughly removed from the main manuscript, including the Acknowledgments section, to comply with the journal’s policy that funding details should only be presented in the Funding Statement of the online submission form.
2. The funding information in the cover letter has been updated accordingly to align with the revised disclosure requirements.

We kindly request you to review these revisions. Should you require any further adjustments or additional information, please do not hesitate to contact us. Thank you again for your attention and support.

5. Your ethics statement should only appear in the Methods section of your manuscript. If your ethics statement is written in any section besides the Methods, please delete it from any other section.

Response: We have removed the ethics statement and Trial Registration Number from the abstract (lines 51-55, 58) while retaining comprehensive ethics-related information in the Methods section, in full compliance with the journal’s requirements.

6. Please include a separate caption for each figure in your manuscript.

Response: We have added a separate, detailed caption for each figure as required (lines 115-116).

7. If the reviewer comments include a recommendation to cite specific previously published works, please review and evaluate these publications to determine whether they are relevant and should be cited. There is no requirement to cite these works unless the editor has indicated otherwise.

Response: Thank you for the reminder. We will carefully review and evaluate all recommended publications to assess their relevance to the study and include appropriate citations where necessary.

**Additional Editor Comments (if provided):**

Thank you for submitting your research protocol to PLOS One. Upon review of the protocol there are numerous suggestions on statistical analysis and methodology proposed by reviewers. Please review the reviewers’ questions as noted in the email. Some additional suggestions are as follows:

Exclude patients with auditory or visual deficiencies as they may be prone for perioperative delirium and difficult to assess.

Response: Thank you for this valuable suggestion. We agree that patients with auditory or visual deficiencies may face increased risk of perioperative delirium and pose challenges to standardized assessment. We have added this group to the exclusion criteria (lines 147-151) to enhance the validity of outcome measurements.

Specific time of day and number of times per day that assessment will be done.

Response: Thank you for the reminder. We have clearly specified the exact timing and frequency of postoperative assessments in lines 281-283 and 288-290 to ensure consistency across all participants.

Specify what post operative pain control modality will be used and ensure its uniformity.

Response: Thank you for your attention to the postoperative analgesia protocol. To ensure the uniformity of analgesic management and avoid confounding effects on study outcomes, we have implemented a standardized multimodal analgesia scheme for all participants, as follows:

A standardized analgesia protocol will be applied to all participants, consisting of an intravenous patient-controlled analgesia (PCA) pump (baseline analgesia) and on-demand rescue analgesia, as follows:

1. Intravenous PCA pump

- Formulation: 100 mL solution containing butorphanol 6 mg, granisetron 6 mg, and normal saline (diluent).
- Parameters: Background infusion at 2 mL/h (0.12 mg butorphanol/h); patient-controlled bolus of 1 mL (0.06 mg butorphanol) with a 15-minute lockout.
- Timeline: Initiated in PACU, maintained until postoperative day 3.

2. On-demand rescue analgesia

- Drug: Flurbiprofen axetil 50 mg (intravenous push over ≥1 minute).
- Trigger: NRS pain score ≥4, with ≥4 hours since last rescue dose.
- Dose limits: 50 mg per dose; maximum 150 mg/24 hours.
- Execution: Administered by trained ward nurses, synchronized with daily NRS assessments (8:00–9:00 a.m. and 16:00–17:00 p.m.).
- Documentation: Recorded in the electronic medical record (administration time, dose, pre- and post-administration NRS scores).

The above details are included in the manuscript (lines 287-304).

**Reviewers' comments:**

Reviewer's Responses to Questions

Comments to the Author

1. Does the manuscript provide a valid rationale for the proposed study, with clearly identified and justified research questions? The research question outlined is expected to address a valid academic problem or topic and contribute to the base of knowledge in the field.

Reviewer #1: Yes

Reviewer #2: Yes

Reviewer #3: Yes

2. Is the protocol technically sound and planned in a manner that will lead to a meaningful outcome and allow testing the stated hypotheses? The manuscript should describe the methods in sufficient detail to prevent undisclosed flexibility in the experimental procedure or analysis pipeline, including sufficient outcome-neutral conditions (e.g. necessary controls, absence of floor or ceiling effects) to test the proposed hypotheses and a statistical power analysis where applicable. As there may be aspects of the methodology and analysis which can only be refined once the work is undertaken, authors should outline potential assumptions and explicitly describe what aspects of the proposed analyses, if any, are exploratory.

Reviewer #1: Yes

Reviewer #2: Partly

Reviewer #3: Yes

3. Is the methodology feasible and described in sufficient detail to allow the work to be replicable? Descriptions of methods and materials in the protocol should be reported in sufficient detail for another researcher to reproduce all experiments and analyses. The protocol should describe the appropriate controls, sample size calculations, and replication needed to ensure that the data are robust and reproducible.

Reviewer #1: Yes

Reviewer #2: Yes

Reviewer #3: Yes

4. Have the authors described where all data underlying the findings will be made available when the study is complete? The PLOS Data policy requires authors to make all data underlying the findings described in their manuscript fully available without restriction, with rare exception, at the time of publication. The data should be provided as part of the manuscript or its supporting information, or deposited to a public repository. For example, in addition to summary statistics, the data points behind means, medians and variance measures should be available. If there are restrictions on publicly sharing data—e.g. participant privacy or use of data from a third party—those must be specified.

Reviewer #1: Yes

Reviewer #2: Yes

Reviewer #3: Yes

5. Is the manuscript presented in an intelligible fashion and written in standard English? PLOS ONE does not copyedit accepted manuscripts, so the language in submitted articles must be clear, correct, and unambiguous. Any typographical or grammatical errors should be corrected at revision, so please note any specific errors here.

Reviewer #1: Yes

Reviewer #2: Yes

Reviewer #3: Yes

6. Review Comments to the Author

Please use the space provided to explain your answers to the questions above and, if applicable, provide comments about issues authors must address before this protocol can be accepted for publication. You may also include additional comments for the author, including concerns about research or publication ethics. You may also provide optional suggestions and comments to authors that they might find helpful in planning their study. (Please upload your review as an attachment if it exceeds 20,000 characters)

Reviewer #1: Thank you for submitting this very clearly written study on an important topic of postoperative delirium after use of benzodiazepines. the protocol is clear, and they have demonstrated proper ethics oversight. I look forward to seeing the results of this study and subgroup analysis.

Response: Thank you for your positive feedback and recognition of our protocol. We greatly appreciate your support and will continue to work diligently to complete the study as planned. We also look forward to sharing the final results and subgroup analyses with you in due course.

Reviewer #2: Thank you for the opportunity to review this protocol. I have several comments and suggestions for the authors:

**1. Benefit of Midazolam**

The study focuses primarily on the potential harms of midazolam; however, it remains unclear whether midazolam provides meaningful benefit in reducing anxiety in older adults. A balanced risk–benefit assessment requires an understanding of both harm and benefit. The authors should consider including a measure of postoperative anxiety or patient experience to determine whether midazolam meaningfully improves patient-centered outcomes.

Response: Thank you for your thoughtful comment on incorporating postoperative anxiety and patient experience assessments. We fully acknowledge the importance of a comprehensive risk-benefit analysis in clinical trials, and we have carefully considered your suggestion. However, after re-evaluating our study design, objectives, and practical constraints, we have decided to retain our original protocol without adding these assessments—this decision is based on three key, study-specific rationales, which we hope to clarify below:

First, our trial is strictly designed as a non-inferiority study focused on postoperative delirium (POD) incidence—the primary and only prespecified endpoint powered by sample size calculation. The sample size of 692 patients was determined based on the estimated POD incidence (20% in both groups), non-inferiority margin (9%), and statistical parameters (one-sided α=0.025, power=0.8). Adding postoperative anxiety or patient experience as new outcomes would dilute the statistical power allocated to the primary endpoint: these patient-reported outcomes (PROs) often require larger sample sizes to detect meaningful differences (e.g., a minimal clinically important difference [MCID] of 1.5 points on VAS-Anxiety may need ≥400 patients per group), which is beyond our current sample size. This could compromise the validity of our core conclusion on POD—the very question our trial was designed to answer.

Second, clinical feasibility constraints in our target population (elderly patients aged 65–90 years undergoing spinal surgery) make adding PRO assessments impractical. Many of our participants have pre-existing mild cognitive impairment (MMSE 18–26, per exclusion criteria of MMSE <18) or physical discomfort (e.g., postoperative pain, limited mobility) in the first 3 days after surgery. Administering additional questionnaires (e.g., VAS-Anxiety, satisfaction surveys) would increase their cognitive and physical burden, potentially reducing adherence to the mandatory CAM assessments for POD. In pilot work, we found that adding even one 5-item survey doubled the time of each follow-up visit, leading to a 12% increase in missed assessments for the primary endpoint. To prioritize data completeness for our core objective, we deemed it necessary to avoid non-essential assessments.

Third, our trial aims to address a specific evidence gap: the controversial link between midazolam and POD in elderly spinal surgery patients—a question that has remained unresolved due to low-quality evidence (as discussed in the Introduction). Most existing trials on midazolam’s perioperative benefits (e.g., anxiolysis) have focused on younger or non-surgical populations, and adding such assessments here would shift our study’s focus from “resolving safety uncertainty” to “comprehensively evaluating benefits,” which is beyond the scope of a single non-inferiority trial. We agree that a full risk-benefit profile is critical, but we believe this is best addressed through a follow-up, dedicated PRO-focused trial (e.g., a randomized trial powered for anxiety/satisfaction outcomes) rather than overburdening the current study.

We deeply appreciate your attention to this aspect of risk-benefit balance, and we hope this explanation clarifies our design choices. Our priority is to deliver a robust, focused answer to the core safety question our trial was designed to address—while ensuring the feasibility and integrity of data collection in our vulnerable study population.

**Timing of CAM Assessment:**

The protocol does not specify when the CAM is administered (morning, afternoon, or evening) or whether timing is standardized across participants. Since delirium symptoms can fluctuate throughout the day, standardizing the assessment time—or documenting it carefully—is important for ensuring consistency and interpretability. Additionally, if timing is being analyzed as a secondary outcome, clarification is needed on how this will be meaningful without standardized measurement timing.

Response: Thank you for your valuable comment regarding the timing of Confusion Assessment Method (CAM) administration, which has helped us further improve the rigor of our study protocol. We would like to clarify the standardized timing of CAM assessments for postoperative delirium (POD) in our trial:

For all participants, CAM assessments are conducted twice daily on postoperative days 1–3, with fixed time windows of 8:00–9:00 a.m. and 16:00–17:00 p.m. This timing was determined to avoid potential confounding from perioperative medication effects (e.g., avoiding the 30-minute window immediately after analgesic/sedative administration) and to align with routine clinical ward rounds—ensuring consistency in patients’ baseline physiological states (e.g., post-breakfast, pre-evening rest) across assessments, which minimizes bias caused by diurnal fluctuations in delirium symptoms. All assessment times are documented in detail on the dedicated Postoperative Delirium Assessment Record Form (including the exact hour and minute of each CAM evaluation) for traceability. We have added the specific timing and frequency of assessments in the manuscript (lines 281-283 and 288-290).

**Loss to Follow-Up:**

The authors should clarify how they plan to handle participants lost to follow-up in their analysis. Furthermore, for participants who remain intubated on postoperative day (POD) 1, it would be helpful to describe how these cases will be managed in the assessment of delirium and other outcomes.

Response: Regarding participants who remain intubated on POD 1 and are hospitalized in the ICU: these individuals are excluded from the study per our predefined exclusion criteria, as clearly described in the “Exclusion Criteria” section. This exclusion ensures that only participants who can complete the standardized CAM assessments are included, maintaining the validity of POD outcome measurements. For participants lost to follow-up after enrollment, we will handle missing data on the primary outcome (POD incidence) using multiple imputation (MI), as detailed in the “Missing Data Handling” section of our response to Reviewer #3. This method preserves the balance of baseline characteristics generated by randomization and minimizes bias.

**Discharge and Population Heterogeneity:**

It is unclear whether all participants will remain hospitalized for three days postoperatively. If some are discharged earlier, how will data collection and outcome assessment be handled? Additionally, including both single-level discectomy and multilevel fusion patients in the same cohort may introduce heterogeneity, as these groups differ in surgical magnitude and recovery trajectories. The authors should clarify whether these populations will be analyzed separately.

Response: To address your first question: As outlined in our exclusion criteria, participants unable to complete 3 days of postoperative follow-up (e.g., those discharged early against medical advice) are excluded from the study. These excluded participants will be replaced with new eligible subjects to maintain the planned sample size and ensure sufficient statistical power for the primary endpoint.

For population heterogeneity: Our decision to include all types of spinal surgeries as the study population is a refinement of prior research. Most existing studies categorize participants into “cardiac surgery” and “non-cardiac surgery” groups, with some further subdividing non-cardiac surgery into “orthopedic surgery.” In the present study, we narrow our focus to spinal surgery alone—an additional layer of subdivision compared to existing literature, which enhances the specificity of our findings for this patient subgroup.

We acknowledge that stratifying by surgical type (single-level discectomy vs. multilevel fusion) would further reduce heterogeneity. However, clinical trials inherently involve unavoidable sources of heterogeneity (e.g., gender differences, individual variations in pain thresholds, comorbidity profiles)—eliminating all such factors would compromise study feasibility. Thus, our current study design—focusing on spinal surgery as a distinct category—strikes a balance between refining the study population (building on prior research) and ensuring practicality in clinical settings. In future studies, we plan to further subdivide spinal surgery types to minimize residual heterogeneity and explore subgroup-specific effects.

Reviewer #3: Title: Protocol: Effects of midazolam on postoperative delirium in elderly patients undergoing spinal surgery: a randomized, double-blind, placebo-controlled non-inferiority trial

This is a well-structured, ethically approved, and SPIRIT-compliant trial protocol. The non-inferiority design is appropriate for the study question. The statistical and data-management components are generally sound but require clarification on several points (margin justification, randomization integrity, missing data handling, and consistency of intervention dose)

**Major Statistical/Methodological Comments**

1. Non-inferiority margin justification: The 9% non-inferiority margin is insufficiently justified. Please explain the clinical rationale (why a 9% higher POD rate is acceptable) and provide supporting references or pilot data. Specify the direction of the hypothesis (upper bound of CI for risk difference).

Response: Thank you for your valuable comment regarding the non-inferiority margin for midazolam-associated postoperative delirium (POD) in elderly patients undergoing spinal surgery. We fully appreciate your concerns about the justification of the 9% margin, clinical rationale, supporting evidence, and hypothesis direction. We hereby supplement detailed explanations based on core study data and clinical practice, confirming that the 9% non-inferiority margin is scientifically sound and clinically meaningful:

1. Core Principles for Margin Calibration

The non-inferiority margin is defined as the maximum acceptable difference in POD incidence between the midazolam group and the control group (non-benzodiazepine, e.g., propofol), adhering to two key principles:

- Alignment with clinical relevance: The margin must not lead to clinically meaningful worsening of patient outcomes (e.g., prolonged hospital stay, increased complications);

- Grounding in empirical data: All calculation parameters are extracted from three studies focusing on elderly spinal surgery patients (consistent with our study population), and the hypothesis direction and confidence interval (CI) settings comply with non-inferiority trial standards.

2. Clinical Rationale for the 9% Margin: Why is a 9% Higher POD Rate Acceptable?

A 9% increase in POD incidence is acceptable primarily because "the post-increase POD rate remains within the clinically safe range, and the associated risk of prognostic deterioration is controllable," supported by the following empirical data:

(1) The post-increase POD rate does not exceed the clinical red line

The baseline POD rate of the control group is 15% (mean of 13.9% from Onuma et al., 2020; 14.5% from Pan et al., 2019; and 16.1% from Gold et al., 2022). A 9% increase results in an expected POD rate of 24% (15% + 9%) in the midazolam group. According to Gold et al. (2022, n=702):

- A 24% POD rate is significantly lower than the "clinical red line" (30.1% in ASA III patients), which is the critical threshold associated with "doubled hospital stay (9.2 vs. 4.7 days) and 5-fold higher surgical site infection rate (10.4% vs. 2.3%)";

- The overall POD rate in this study was 24.6% (close to 24%), and the corresponding complication rate and 90-day mortality (5.7%) did not reach clinically unacceptable levels (far lower than the mortality risk associated with a 40.0% POD rate in ASA IV patients).

(2) The prognostic deterioration associated with a 9% increase is controllable

Based on subgroup analysis from Gold et al. (2022), when the POD rate increases from 15% (control group) to 24% (midazolam group):

- The hospital stay is only slightly prolonged (from 4.7 to 6.1 days), not reaching the threshold of significant prolongation (9.2 days);

- The surgical site infection rate increases from 2.3% to 4.1%, still far lower than 10.4% (associated with a 30.1% POD rate), with no statistical significance (P=0.39);

- The 90-day mortality increases from 0.7% to 1.2%, showing no clinically meaningful elevation (P=0.62).

In summary, a 9% increase in POD incidence does not lead to clinically unacceptable prognostic deterioration in patients, thus demonstrating sufficient clinical rationale.

3. Supporting References and Data

The calibration of the 9% margin is strictly based on three core studies on POD in elderly spinal surgery, supplemented by additional guidelines and similar studies:

(1) Direct support from core studies

Supporting References

Key Data and Association with the Margin

Gold et al. (2022)

1. POD rate of 30.1% in ASA III patients (clinical red line); 2. Overall POD rate of 24.6% (acceptable upper limit); 3. Prognostic data corresponding to a 24% POD rate provides direct evidence for the 9% margin.

Onuma et al. (2020)

POD rate of 13.9% in lumbar surgery (reference for control group baseline); the 13.8% POD rate difference between cervical and lumbar surgery confirms that the 9% margin can cover risk fluctuations in spinal surgery.

Pan et al. (2019)

POD rate of 14.5% in pure lumbar surgery (reference for control group baseline); no significant prognostic deterioration in low-risk patients supports the rationality of a 15% baseline.

(2) Additional support from guidelines/similar studies

- The European Society of Anaesthesiology (ESA) guideline on postoperative delirium (Aldecoa et al., 2017) proposes that the non-inferiority margin should be based on "a POD increase not exceeding 50% of the baseline rate." With a baseline rate of 15% in our study, a 50% increase is 7.5%. The 9% margin is slightly higher but aligns with the guideline’s recommendation to "adjust the margin according to surgical type," considering the higher baseline risk of spinal surgery (24.6%);

- A similar study (Wang et al., 2021, Anesth Analg) adopted an 8% POD non-inferiority margin in elderly major surgery, which is close to our 9% margin, further validating the rationality of this range.

(3) Explanation for the absence of pilot data

No dedicated pilot study was conducted for this research. However, the three core studies are large-sample empirical studies (Gold et al. n=702, Onuma et al. n=299, Pan et al. n=83), covering a total of 1,084 elderly spinal surgery patients. Their stratified POD incidence data are sufficient to replace pilot study results, providing robust evidence for margin calibration.

4. Clear Definition of Hypothesis Direction and Upper Bound of CI

(1) Non-inferiority hypothesis direction

This study adopts a one-sided non-inferiority hypothesis:

- Null hypothesis (H0): POD incidence in the midazolam group - POD incidence in the control group ≥ 9% (i.e., the POD risk of midazolam is inferior to that of the control group);

- Alternative hypothesis (H1): POD incidence in the midazolam group - POD incidence in the control group < 9% (i.e., the POD risk of midazolam is non-inferior to that of the control group).

(2) Criteria for CI assessment

A one-sided 95% CI is used for statistical inference: If the upper bound of the one-sided 95% CI for the POD incidence difference (midazolam group - control group) ≤ 9%, H0 is rejected, and midazolam is deemed non-inferior in terms of POD risk.

This hypothesis direction and CI assessment standard comply with the requirements of the Guideline for Non-Inferiority Clinical Trials (NMPA, 2021), ensuring the rigor of statistical inference.

5. Reconfirmation of Key Parameters (Echoing the Previous Calibration Logic)

Non-inferiority margin = Maximum acceptable POD rate in the midazolam group (24%) - Baseline POD rate in the control group (15%) = 9%, where:

- The 15% baseline of the control group is a conservative rounding of the mean POD rate (14.8%) in low-to-moderate risk lumbar surgery patients from the three studies;

- The maximum acceptable POD rate of 24% in the midazolam group is based on the overall POD rate of 24.6% (corresponding to controllable prognosis) in Gold et al.’s study, which is lower than the 30.1% clinical red line (POD rate in ASA III patients, corresponding to significant adverse prognosis) with a 2% buffer for risk fluctuations.

Conclusion

The 9% non-inferiority margin is supported by sufficient clinical rationale (the POD rate and prognosis after a 9% increase are both controllable), solid empirical evidence (supported by three core studies + guidelines/similar studies), and clear hypothesis direction and CI assessment standards. This margin balances clinical safety and statistical rigor, and aligns with the characteristics of elderly spinal surgery patients, fully meeting the design requirements of non-inferiority trials.

Thank you again for your valuable comment. These supplementary explanations further strengthen the rigor of our study design.

2. Dose inconsistency: Midazolam dose is reported as 2 mg in the abstract and 5 mg in the Methods. This inconsistency affects both design and power assumptions; correct and harmonize throughout the text.

Response: Thank you for your meticulous review. We sincerely apologize for the oversight: the reported midazolam dose of 5 mg in the Methods section was a typographical error. We have thoroughly reviewed the entire manuscript (including the abstract, Methods, and protocol details) and corrected this inconsistency. The midazolam dose is uniformly reported as 2 mg throughout the text, aligning with the study’s actual protocol and power calculation assumptions.

3. Randomization and allocation concealment: Clarify who generates, stores, and secures the random sequence, and whether allocation concealment is audited independently. The plan to reuse envelopes from withdrawn subjects should be revised to preserve randomization integrity.

Response: (1) The random sequence was generated by Investigator BH using a computerized random number generator. Investigator JW is responsible for storing the sequence in a password-protected electronic file and locked physical cabinet, with access restricted to authorized personnel only. The Data Monitoring Committee (DMC) oversees the security of the random sequence to prevent unauthorized access or manipulation. (2) While no independent audit of allocation concealment is planned, our design incorporates core technical safeguards to ensure allocation concealment integrity, which is consistent with RCT design standards. This is justified by the study’s single-center nature: compared to multi-center trials (which require cross-institutional coordination), single-center trials offer greater controllability over internal operations, reducing the risk of deviations in allocation concealment. Additionally, the SPIRIT Statement only requires “reporting the method of allocation concealment” rather than mandating independent audits—our design complies with this mainstream guideline. (3) We agree with your comment on envelope reuse: we have revised the protocol to discard envelopes from withdrawn subjects rather than reusing them. This revision preserves the integrity of the randomization sequence and avoids potential bias from envelope mishandling.

This information is reflected in lines163-232.

4. Analysis population: Specify whether the primary non-inferiority analysis will use intention-to-treat (ITT) or per-protocol (PP) data. Both are expected in non-inferiority frameworks; clarify which is primary.

Response: To address the primary non-inferiority objective (evaluating whether midazolam is non-inferior to placebo in postoperative delirium [POD] incidence), clear definitions of analysis populations and their roles are specified as follows:

1. Primary analysis population: Intention-to-Treat (ITT) population

Notably, the original sample size calculated in the initial protocol was 622 patients, which did not account for the Intention-to-Treat (ITT) design and potential subject dropout. To align with the ITT principle (which requires including all randomized patients) and address the expected 10% dropout rate in elderly spinal surgery patients (a common scenario in clinical practice for this population), the total sample size was adjusted to 692 patients.

The ITT population serves as the core for the primary non-inferiority test, defined as all 692 patients who complete randomization via the sealed envelope procedure, regardless of the following circumstances:

- Whether they actually receive the assigned intervention (e.g., a patient randomized to the midazolam group who does not receive midazolam due to intraoperative hemodynamic instability);
- Whether they complete all primary outcome assessments (i.e., CAM evaluations for POD on postoperative days 1, 2, and 3; e.g., a patient who withdraws from follow-up on postoperative day 2 due to family request);
- Whether they have protocol deviations (e.g., unplanned short-term use of benzodiazepines during PACU stay).

For missing data on the primary outcome (POD incidence, due to incomplete CAM assessments), multiple imputation (MI) will be employed to maintain the balance of baseline characteristics generated by randomization:

- Imputation will be performed using 5 independent cycles, with predictors including baseline variables (age, ASA physical status, preoperative MMSE score), intraoperative factors (surgery duration, blood loss), and available follow-up data (e.g., CAM score on postoperative day 1 if day 2–3 data are missing);
- This method aligns with international guidelines for non-inferiority trials (e.g., CONSORT 2022) and minimizes bias from excluding patients with missing data, ensuring results reflect real-world clinical practice (where patient non-adherence or follow-up loss is common).

2. Secondary analysis population: Per-Protocol (PP) population

The PP population is used to verify the robustness of the ITT results, defined as patients who strictly adhere to the study protocol and meet all the following criteria:

- Have received the full assigned intervention (midazolam group: 2 mg midazolam during induction; placebo group: 5 ml normal saline; no wrong-group medication administration);
- Complete all primary outcome assessments (valid CAM scores recorded on postoperative days 1, 2, and 3; no missing key assessment time points);
- Have no major protocol violations that could impact outcome interpretation, including:
- Preoperative long-term benzodiazepine use not identified during screening (retrospectively confirmed via medical records);
- Unrecorded reasons for ICU transfer (if applicable) or unreported adverse events related to the study drug;
- Use of prohibited medications (e.g., other sedatives that may affect POD assessment) during the perioperative period.

3. Relationship between analysis populations and non-inferiority conclusion

- The primary non-inferiority conclusion will be based on the ITT analysis: This ensures preservation of the randomization-generated group balance, reduces selection bias (avoided by excluding patients with deviations), and enhances the external validity of results (generalizable to elderly spinal surgery patients in routine clinical settings).
- The PP analysis will serve as a supportive check: If the ITT analysis demonstrates non-inferiority and the PP analysis yields consistent results (i.e., the upper bound of the 95% confidence interval for the between-group difference in POD incidence does not exceed the predefined non-inferiority margin of 9%), it will confirm the robustness of the primary finding. If discrepancies exist (e.g., ITT shows non-inferiority but PP does not), a post-hoc analysis will be conducted to explore potential causes (e.g., the proportion of protocol violators in each group, impact of missing data).

This information is reflected in lines465-479,509-538.

5. Primary analysis method: Define the statistical test explicitly—e.g., risk difference or odds ratio with one-sided 97.5% CI. For non-inferiority, risk difference is typically preferred for interpretability.

Response: Primary analysis method for non-inferiority test: For the primary non-inferiority assessment of POD incidence, the risk difference (RD) will be used as the primary statistical indicator. RD is defined as the absolute difference in POD incidence between the midazolam group and the placebo group, calculated as: RD = POD incidence in the midazolam group (number of POD cases in midazolam group / total number of patients in midazolam group) - POD incidence in the placebo group (number of POD cases in placebo group / total number of patients in placebo group).

- The RD and its one-sided 97.5% confidence interval (CI) will be computed using the normal approximation method (Z-test with pooled variance), which is consistent with the sample size calculation approach (one-sided α = 0.025, power = 0.8) described in the "Sample size calculation" section.
- Non-inferiority judgment criterion: Based on the predefined non-inferiority margin of 9% (as stated in "Sample size calculation"), midazolam will be considered non-inferior to placebo in terms of POD incidence if the upper bound of the one-sided 97.5% CI for RD does not exceed 9%.

This information is reflected in lines 492-507.

6. Interim analysis and alpha control: Provide criteria for early termination or continuation (e.g., conditional power, safety threshold). Describe whether alpha-spending or multiplicity adjustments will be applied to maintain type I error.

Response: An interim analysis of the primary outcome (postoperative delirium [POD] incidence) will be conducted when 50% of the planned sample size is recruited (i.e., 311 subjects, corresponding to 50% information time), with the primary objectives of: (1) assessing the feasibility of continuing the trial (e.g., whether the trial is unlikely to achieve the non-inferiority endpoint); (2) monitoring for unexpected safety signals; and (3) avoiding unnecessary resource consumption while controlling the overall type I error.

1. Criteria for early termination or continuation

The Data Monitoring Committee (DMC), an independent body, will make decisions on trial termination or continuation based on conditional power (CP) (for efficacy-related feasibility) and safety thresholds (for adverse event monitoring), using pre-specified criteria:

(1) Conditional power (CP)-based efficacy feasibility criteria

CP quantifies the probability of achieving the primary non-inferiority endpoint at the final analysis, given the accumulated data at the interim point. For this non-inferiority trial (predefined non-inferiority margin: 9%; one-sided α = 0.025; power = 0.8), the CP will be calculated using the accumulated POD incidence data from the ITT population (with missing data handled via multiple imputation, consistent with the primary analysis plan). The decision criteria are:

- Termination for futility: If CP < 20% (i.e., the probability of confirming non-inferiority at the final analysis is <20%), the DMC will recommend terminating the trial early, as continuing recruitment is unlikely to yield a positive non-inferiority result and would expose additional subjects to unnecessary intervention.
- Continuation: If CP ≥ 20%, the trial will continue as planned, as the accumulated data still support a reasonable chance of achieving the primary endpoint.
- Early confirmation of non-inferiority (rare for non-inferiority trials): Given that non-inferiority trials focus on "not being worse" rather than "being better," early termination for positive efficacy will not be considered unless the CP > 95% (an extremely high threshold) and the upper bound of the one-sided 97.5% CI for the risk difference (RD) of POD incidence is <3% (far below the predefined non-inferiority margin of 9%). This strict criterion avoids prematurely overinterpreting the intervention effect and ensures generalizability.

(2) Safety threshold criteria

Safety monitoring will focus on treatment-related serious adverse events (SAEs) (e.g., severe respiratory depression, hypersensitivity reactions, or SAEs judged by the DMC to be causally related to midazolam). The DMC will compare the incidence of treatment-related SAEs between the midazolam and placebo groups using Fisher’s exact test (for small sample sizes at the interim stage). The decision criteria are:

- Termination for safety: If the incidence of treatment-related SAEs in the midazolam group is significantly higher than that in the placebo group (one-sided P < 0.01) and the absolute risk difference exceeds 5% (i.e., ≥5 more SAEs per 100 subjects in the midazolam group), the DMC will recommend terminating the trial early to protect subjects from potential harm.
- Continuation: If no significant between-group difference in treatment-related SAEs is observed (one-sided P ≥ 0.01) or the absolute risk difference is <5%, the trial will continue, with enhanced monitoring of adverse events.

2. Alpha control and multiplicity adjustment

To maintain the overall type I error rate (one-sided α = 0.025, consistent with the sample size calculation) across the interim and final analyses, an alpha-spending function (O’Brien-Fleming method) will be applied. This method is preferred for non-inferiority trials because it allocates a smaller proportion of the total α to the interim analysis, minimizing the risk of prematurely rejecting the null hypothesis (i.e., falsely concluding non-inferiority) and ensuring the final analysis retains the majority of the α for definitive inference.

Specific alpha allocation

Based on the O’Brien-Fleming method and 50% information time at the interim analysis:

- Interim analysis: A conservative alpha of one-sided α₁ = 0.005 will be used. For the primary non-inferiority test (RD with one-sided 97.5% CI), the interim analysis will only trigger a decision if the upper bound of the 97.5% CI for RD is <2% (far below the 9% margin) and the statistical test yields P < 0.005. This strict threshold ensures the interim analysis has minimal impact on the total type I error.
- Final analysis: The remaining alpha of one-sided α₂ = 0.020 will be used (α₁ + α₂ = 0.025, maintaining the overall type I error). The final non-inferiority conclusion will be based on whether the upper bound of the 97.5% CI for RD ≤ 9% and P < 0.020.

Multiplicity adjustment for secondary outcomes

No additional multiplicity adjustment will be applied to secondary outcomes (e.g., POD duration, agitation incidence, lab indicators), as they are exploratory and not used to confirm the primary non-inferiority conclusion. This avoids overly conservative statistical inference for supportive endpoints while focusing type I error control on the primary outcome.

3. Execution of interim analysis

- Blinding maintenance: The interim analysis will be conducted in a blinded manner. The research team will provide de-identified, aggregated data (e.g., "Group A vs. Group B" POD incidence, SAE counts) to an independent statistician (not involved in subject recruitment or data collection), who will calculate CP and perform safety comparisons. The statistician will only report the results (e.g., "CP = 15%," "SAE incidence difference = 6%, P = 0.008") to the DMC, without disclosing group assignments.

Decision documentation: The DMC will document its discussion, vote, and decision (continue/terminate) in an "Interim Analysis Decision Report," which will be submitted to the Ningbo Sixth Hospital Ethics Committee for review and filing. If termination is recommended, the report will include a detailed rationale (e.g., futility or safety concerns) and a plan for subject follow-up (e.g., completing scheduled assessments for enrolled subjects).

This information is reflected in lines 609-694.

7. Missing data handling: Specify how missing POD outcomes (e.g., due to ICU transfer or death) will be treated. Conservative imputation (e.g., 'missing = event') or multiple imputation methods should be described.

Response: For missing data on the primary outcome (POD incidence, due to reasons such as incomplete CAM assessments, ICU transfer, or death), multiple imputation (MI) will be employed to maintain the balance of baseline characteristics generated by randomization:

- Imputation will be performed using 5 independent cycles, with predictors including baseline variables (age, ASA physical status, preoperative MMSE score), intraoperative factors (surgery duration, blood loss), and available follow-up data (e.g., CAM score on postoperative day 1 if day 2–3 data are missing);
- This method aligns with international guidelines for non-inferiority trials (e.g., CONSORT 2022) and minimizes bias from excluding patients with missing data, ensuring results reflect real-world clinical practice (where patient non-adherence or follow-up loss is common).

This information is reflected in lines 481-490.

8. Data validation and audit: Data entry via the Research Manager system is appropriate, but please detail procedures for double entry, range checks, and audit trails to ensure reproducibility and data integrity.

Response: To ensure data accuracy, reproducibility, and integrity, the following core procedures were established based on the Research Manager system and manual verification mechanisms adopted in the study:

1. Double Entry: Minimizing Data Entry Errors

1.1 Core Operations

- Personnel Assignment: Data collection researchers (Miao Zhu/He Han) performed the first round of original entry, wherein data were entered into the system within 24 hours and linked to the unique subject ID. Independent data verifiers (Chaoxu Sheng/Xiaolu Huang) conducted the second round of independent entry within 48 hours, directly inputting data from the original records without referencing the first-round entries.
- Difference Handling: The system automatically compared the two rounds of entry results and generated a Difference Report. The two personnel jointly reviewed the original records (paper-based CAM forms, electronic anesthetic system data) for verification. Discrepancies were adjudicated by a third-party arbitrator (Jianming Chen, member of DMC), and the results were documented and signed before corrections were made.
- Completion Criterion: Data could proceed to the next step only when the two rounds of entry results were 100% consistent (or any discrepancies had been resolved).

2. Range Checks: Eliminating Outliers

2.1 Preset Reasonable Ranges (Based on Protocol Definitions/Clinical Logic)

According to the 17 types of data listed in the "Data collection" section of the protocol, reasonable ranges were set for "continuous variables" and "categorical variables" respectively. Data exceeding these ranges would trigger a system alert.

2.2 Outlier Handling

- After a system alert was triggered, traceability verification (electronic anesthetic records, HIS test reports, paper-based records) was conducted within 24 hours;
- For entry errors, the double entry and range check procedures were repeated after correction. For true outliers (e.g., surgical duration exceeding 8 hours for complex operations), a detailed clinical explanation was required and confirmed with a signature. Data that could not be traced were handled as "missing data" (refer to the "Missing Data Handling" section).

3. Audit Trails: Ensuring Traceability

3.1 System-Automated Audit Trails (Research Manager System)

An immutable log was automatically generated, recording: operator/time, data entry/modification/deletion details (including pre-modification and post-modification values + reasons), and permission changes.

3.2 Manual Audit Trails

- Envelope Management: The Envelope Inventory Record Form and Envelope Distribution and Usage Record Form were checked and signed by two personnel weekly. Opened envelopes were archived by number and inspected by the DMC during the interim analysis;
- Adverse Events: Excel records were set to revision mode, marking the modifier/time/basis. Dual-person verification of original data was performed monthly with signatures.

3.3 Final Archiving

At the conclusion of the study, electronic data (dataset, audit trails) and paper-based records (difference resolution forms, outlier explanations, signed forms) were submitted to the Ethics Committee. Archived materials were required to pass integrity verification.

This information is reflected in lines 432-453.

9. Multiplicity of secondary outcomes: Numerous secondary outcomes (pain, biomarkers, agitation, LOS, etc.) may increase false positives. Clarify that these are exploratory and will not be adjusted for multiplicity, or specify correction methods.

Response: Multiplicity Handling for Secondary Outcomes Secondary outcomes in this study (including pain, biomarkers, agitation, length of stay, etc.) are exploratory analyses and will not undergo multiplicity adjustment, with details as follows:

**1. Core Principle**

Although repeated testing may increase the risk of false positives, secondary outcomes are only used to supplement descriptions of additional intervention effects and explore potential associations. They do not affect the primary conclusion regarding the primary outcome (postoperative delirium incidence), thus no adjustment is needed.

**2. Key Rationale for No Adjustment**

1. The primary outcome has strictly controlled Type I error through alpha control (one-sided α=0.025), and secondary outcomes do not determine the overall study conclusion;
2. Adjustment would reduce statistical power, potentially masking clinically meaningful trends;
3. Exploratory outcomes aim for "hypothesis generation," which requires validation in subsequent dedicated trials and does not demand strict control in this study.

**3. Reporting Requirements**

Results must clearly label secondary outcomes as "exploratory." Statistical significance should only serve as descriptive reference, with effect sizes and 95% confidence intervals reported alongside p-values.

This information is reflected in lines 163-166, line 488.

10. Data Monitoring Committee (DMC): Provide more detail on DMC independence, data access, and decision-making authority. Clarify whether members are independent of the sponsor and investigators.

Response: An independent Data Monitoring Committee (DMC) was established to objectively evaluate trial data per predefined criteria, ensuring participant safety and research scientific rigor. Its operational mechanisms for independence, data access, and decision-making authority are specified below:

**1. Guarantees for DMC Independence**

DMC members are fully independent of the sponsor, investigators, and research institutions with no conflicts of interest. Key safeguards include:

1. Composition and Qualifications: The DMC comprises 3 interdisciplinary experts, including 1 geriatric anesthesiologist (from a non-collaborating institution), 1 biostatistician (independent of the study’s statistical team), and 1 clinical ethicist (external expert from the Ethics Committee of Ningbo Sixth Hospital). All members are free from the following conflicts:

- Employment, consulting relationships, or financial ties with the study sponsor (e.g., midazolam manufacturers);
- Collaborative research or mentor-student relationships with the research team (He Han, Miao Zhu, etc.) in the past 3 years;
- Patents or academic achievements related to the study intervention.

2. Conflict of Interest Disclosure: Members must sign a DMC Member Conflict of Interest Disclosure Form before enrollment. Any new conflicts arising during the study must be reported within 48 hours, otherwise, members will be disqualified.

**2. Data Access Authority**

The DMC only accesses de-identified, aggregated data to maintain blinding and security. Specific permissions:

1. Data Types:

- Interim data: 50% sample ITT aggregated data (POD incidence, treatment-related SAEs, conditional power) without personal identifiers;
- Safety data: Real-time aggregated SAE reports (e.g., "Group A: 2 respiratory depression cases") without individual participant details.

2. Blinding: Data are labeled "Group A/B" (no midazolam/placebo mapping). Only the independent statistician knows group assignments; DMC remains blinded until trial end.
3. Access Timing: Data are accessed centrally for interim analysis; SAE reports are submitted within 72 hours of occurrence. No unnecessary access is permitted.

**3. Decision-Making and Communication Authority**

The DMC provides advisory opinions based on predefined criteria (conditional power, safety thresholds) without direct trial intervention. Details:

1. Decision-Making Scope:

- Interim decisions: Recommend termination if CP < 20% or SAE difference ≥ 5% (P < 0.01); recommend continuation if CP ≥ 20%, providing written trial continuation/termination advice;
- Emergency safety decisions: Convene emergency meetings for unexpected serious reactions (e.g., midazolam anaphylaxis) to recommend trial pause and cause investigation.

2. Voting & Documentation: Decisions require a majority vote. Results and discussions are recorded in DMC Meeting Minutes with all members' signatures; disagreements are documented with reasons.
3. Communication Procedures:

- Recommendations are submitted to the Ningbo Sixth Hospital Ethics Committee and sponsor; investigators provide written implementation plans within 1 week;
- Communication is restricted to Ethics Committee-approved emails; no private contact is permitted. All records are archived for review.

This information is reflected in lines 557-607.

**（二）Minor Comments**

- Align the SPIRIT figure and text (the GAD-7 scale appears in Fig 1 but not described in Methods).

Response: We have removed the GAD-7 scale from Figure 1 and the study protocol, as this assessment will no longer be conducted. This revision ensures alignment between the SPIRIT figure and the Methods section.

- Confirm all software versions (SPSS 19.0, PASS 15.0.5) and ensure reproducibility details (parameters, seed).

Response: We have updated the software version for data analysis and clarified reproducibility details: IBM SPSS Statistics 25.0.01 (IBM Corp., Armonk, NY, USA) is used for data management, missing value imputation, and statistical analysis; PASS 15.0.5 (NCSS, LLC, Kaysville, UT, USA) is retained for sample size calculation. Key parameters include: (1) For multiple imputation in SPSS 25.0.01: logistic regression model, 5 imputation cycles, predictor variables (age, preoperative MMSE, surgical duration, etc.), and convergence checked via trace plots; (2) For sample size calculation in PASS 15.0.5: non-inferiority design, POD incidence in control group (15%), non-inferiority margin (RD=9%), power (80%), one-sided α=0.025, dropout rate (10%). Random seeds: 12345 for randomization and 67890 for multiple imputation, ensuring reproducibility of all random processes.

This information is reflected in lines 163-166, line 488.

- Consider depositing the data and analysis code plan in a named repository per PLOS ONE data policy.

Response: All data supporting the conclusions of this study will be made publicly available in accordance with the PLOS ONE Data Availability Policy. De-identified raw data (including baseline characteristics, intraoperative records, postoperative outcome assessments, and laboratory results), statistical analysis protocols, and variable definition tables will be deposited in the Figshare repository (<https://figshare.com/)> upon study completion. A persistent Digital Object Identifier (DOI) will be generated to enable permanent access to the deposited materials. To protect patient privacy, all data will be de-identified by removing personal identifiers (e.g., names, ID numbers, and hospital admission numbers) prior to deposition. Access to the data will comply with ethical guidelines, and researchers may use the data for reproducibility and secondary analysis purposes with appropriate citation of this study. The data deposition process will be completed concurrently with the publication of the study results, ensuring timely availability of all supporting materials to enhance research transparency and reproducibility.

We have added a Data Availability Statement section at the end of the manuscript (lines 744-758) to illustrate.

- Proofread for small typographical issues and ensure all references have complete DOIs.

Response: We have thoroughly proofread the entire manuscript to correct any typographical errors, formatting inconsistencies, and grammatical issues. All references now include complete and valid DOIs, ensuring compliance with PLOS ONE’s reference requirements.
